# Marginal Blepharitis

**Published:** 1908-11-14

**Authors:** 


### Ophthalmology.

### MARGINAL BLEPHARITIS.

Marginal blepharitis is at the same time one of the most irritating affections of the eye and one of the most difficult to treat. It is comparatively common among the lower classes, though it may occasionally be met with among the children of the well-to-do. It is characterised by redness of the edge of the lids, and the formation of crusts or scales at the roots of the lashes, and is often associated with corneal ulcers and overflow of the tears. If untreated the scales or crusts may cause actual ulceration of the lid margins, and the continual inflammation may lead to disease of the hair follicles and ultimately to the entire loss of the eyelashes. At the same time the conjunctiva on the inner surface of the lid margin may become thickened from the inflammation of the lids and lead to ectropion; this ectropion leads to dryness of the exposed conjunctiva and starts a vicious circle, the conjunctiva as a consequence becoming more and more thickened, and aggravating the ectropion and consequent disfigurement. The hypertrophy of the lid margin may also lead to closure of the puncta lachrymalia and cause the most troublesome epiphora with eczema of the skin of the face in the neighbourhood. Thus marginal blepharitis should be treated as early as possible, however slight it may appear at first.

It may be taken as an axiom that the great majority of cases of blepharitis are due in the first place to low errors of refraction. It is interesting to note that as high degrees of hypermetropia or astigmatism seldom give rise to headache, while low degrees usually cause the most persistent trouble, so marginal blepharitis is usually caused by low refractive errors, the commonest cause being a slight degree of myopic astigmatism. As marginal blepharitis usually affects young children at school, it is essential that the refraction should be tested in all cases, the patient being fully atropinised for a week beforehand. Glasses, if any are required, should be worn constantly, and not only for school work; and directly the glasses have been ordered the local treatment should be begun. In mild cases it is usually found that the glasses alone are almost sufficient to ensure a cure, but if there is much redness and incrustation of the edges of the lids it is advisable to wash these crusts off with a warm alkaline boracic lotion. The following formula is the most usual:—

| | |
|---|---|
| Sodium bicarbonate ... ... ... | gr. vij. |
| Borax ... ... ... ... ... | gr. vij. |
| Sodium chloride ... ... ... ... | gr. vij. |
| Refined sugar ... ... ... ... | gr. xv. |
| Water ... ... ... ... ... | ʒij. |

At the same time the B.P. dilute mercury nitrate ointment may be put upon the edges of the lids in the mornings and carefully wiped off after an hour; it is not advisable to put ointment on the edges of the lids at night.

As alternatives to the above the prescriptions which follow may be found useful.

| | |
|---|---|
| White precipitate of mercury ... ... | gr. ss. |
| Neutral acetate of lead ... .. ... | gr. xlv. |
| Oil of sweet almond ... ... ... | ♏ viij. |
| Vaseline ... ... ... ... ... | ʒ 1¼ |

| | |
|---|---|
| Salicylic acid ... ... ... ... | grs. xv. |
| Oxide of zinc ... ... ... ... | ʒ ijss. |
| Powdered starch ... ... ... | ʒ iv. |
| White vaseline ... ... ... ... | ʒ v. |

In many cases which are comparatively unyielding it is often surprising what good results may be obtained from treating the lids with a 15 per cent. solution of protargol. The lids are kept closed, and a large soft camel's hair brush is dipped in the protargol and the edges of the lids vigorously rubbed with it in the manner of lathering the face before shaving. This is continued for several minutes and the superfluous protargol wiped off with damp wool. The treatment may be repeated daily for a week or ten days, at the end of which time it will be found that the crust formation is much diminished and the edges of the lids are cleaner and dryer. In slight cases of blepharitis there is often considerable itching, and this may be relieved by resorcin ointment, gr. xv. in ʒijss. of vaseline, or carbolic acid gr. iij. in ʒijss. of vaseline.

The more severe cases of blepharitis are to a great extent incurable, as the loss of the lashes is permanent and very disfiguring. Much may be done, however, to alleviate discomfort, and the redness and eczema due to the overflow of tears can be diminished.

The epiphora is due to two causes: in the first place the thickening of the lid edges leads to obstruction of the orifices of the glands which secrete the sebaceous material which under normal circumstances greases the edge of the lids and prevents the overflow of tears. If this grease is absent, the whole edge of the lid becomes wet and the tears overflow more readily. In the second place, as has been mentioned above, the puncta lachrymalia may become occluded and the normal outflow of the tears be lost. Under such circumstances the epiphora is very marked and leads to a constant moisture of the lids and of the skin of the face in the neighbourhood. This last condition is most intractable to ordinary treatment, and unless something radical is done will only get steadily worse. Under these circumstances it is essential that the canaliculi in the lower lids should be slit up right into the lachrymal sac, the incision being carried more to the inner side of the edge of the lid than usual. The normal flow of tears is re-established, and the moisture and eczema of the face and lids rapidly clear up under ordinary remedies. Where there is considerable ectropion owing to hypertrophy of the conjunctiva, it may be necessary to excise the diseased portion or cauterise it before the lid and the puncta lachrymalia can be restored to their proper position of contact with the globe.

In the treatment of marginal blepharitis the first essential of success is to correct any error of refraction. Without this precaution other treatment is merely ploughing the sands.

